# Bullous Pemphigoid as a Manifestation of Graft-Versus-Host Disease Following Allogeneic Hematopoietic Stem Cell Transplantation: A Systematic Review and Report of a Novel Case

**DOI:** 10.3390/jcm14124068

**Published:** 2025-06-09

**Authors:** Sapir Glazer Levavi, Moshe Yeshurun, Pia Raanani, Mor Frisch, Meital Oren-Shabtai, Lev Pavlovsky, Daniel Mimouni, Anna Aronovich

**Affiliations:** 1 Division of Dermatology, Rabin Medical Center, Beilinson Hospital, Petach Tikva 49100, Israelanaronovich@gmail.com (A.A.); 2 Institute of Hematology, Rabin Medical Center, Beilinson Hospital, Petach Tikva 49100, Israel; 3 Faculty of Medical and Health Sciences, Tel Aviv University, Tel Aviv 69978, Israel

**Keywords:** bullous pemphigoid, graft-versus-host disease, hematopoietic stem cell transplantation

## Abstract

**Background/Objective**: Bullous Pemphigoid (BP) is a well-recognized autoimmune subepidermal blistering disease. However, its occurrence following allogeneic hematopoietic stem cell transplantation (HSCT) is extremely rare. The objective of this study is to systematically review the available data on BP following an allogeneic HSCT with focus on treatment options. **Methods**: A systematic review of studies evaluating BP following allogeneic HSCT, incorporating a highly treatment-resistant case from our graft-versus-host disease (GvHD) dermatology clinic, of a 47-year-old patient, notable as the only reported instance of BP following HSCT in a patient with chronic lymphocytic leukemia (CLL) that transformed into diffuse large B-cell lymphoma (DLBCL) and GvHD due to HSCT. The review yielded 15 publications that met the eligibility criteria. Including our case, a total of 16 cases were analyzed. **Results**: Nearly all patients (14/16) in this review had chronic GvHD due to their HSCT. Twelve patients were males, and six were of Japanese origin. The mean age for BP diagnosis was 38 years (a range of 5–67). On average, BP developed one year post-HSCT. The most common treatment for BP in these patients was prednisolone, with the majority experiencing complete resolution of symptoms. **Conclusions**: BP following HSCT is an exceptionally rare condition with an unclear underlying mechanism.

## 1. Introduction

Bullous pemphigoid (BP) is the most common autoimmune subepidermal blistering disease. It typically presents in older adults as a chronic, relapsing–remitting, generalized pruritic bullous eruption and is potentially associated with significant morbidity. In the early pre-bullous stages, urticarial plaques may be present. The diagnosis is based on the presence of a characteristic clinical and histological feature and is supported by immune-serological assays, particularly direct and indirect immunofluorescence microscopy (DIF, IIF), as well as an enzyme-linked immunosorbent assay (ELISA), for anti-BP180/BP230 autoantibodies [1].

BP is associated with tissue-bound and circulating autoantibodies directed against BP antigen 180 (BP180, BPAG2, or type XVII collagen) and BP antigen 230 (BP230 or BPAG1e), components of the hemidesmosomes which are essential for dermal–epidermal adhesion. CD4+ T cells, with a mixed Th1/Th2 cytokine profile, proliferate in response to BP180 peptides, while B cells produce antibodies against BP180 and BP230. Macrophages facilitate blister formation by recruiting neutrophils and promoting proinflammatory crosstalk [2].

Hematopoietic stem cell transplantation (HSCT) involves the administration of healthy hematopoietic stem cells to patients with various hematopoietic malignancies. Several types of HSCT are used clinically, with transplanted cells sourced from various origins. In allogeneic HSCT, donors may be HLA-matched relatives, haploidentical family members, or unrelated individuals matched for the human leukocyte antigen (HLA) [3]. HSCT may be complicated by graft-versus-host disease (GvHD) which arises when donor T lymphocytes recognize the recipient tissues as foreign because of histocompatibility differences and initiate an immune response [4].

Previous studies demonstrated an important role of the B-cell immune response in chronic GvHD. Donor B cells function as antigen-presenting cells, promoting the survival and proliferation of pathogenic CD4+ T cells, which are essential for the development of chronic GvHD [5]. Furthermore, chronic GvHD B cells produce circulating antibodies against exposed basement membrane components, such as collagen VII, BP230, BP180, and laminin γ1 in response to GvHD keratinocyte damage and reduced self-tolerance [6]. The dysregulated CD4+ T cells and circulating autoantibodies may contribute to BP formation in patients with GvHD. Some researchers have speculated that HLA types or micro-chimerism may also play a role in autoimmune blistering dermatosis [7].

Reports on BP following solid organ transplantation or HSCT are scarce [8], and the pathogenesis remains unclear. We systematically reviewed the literature on the prevalence, characteristics, and management of BP following HSCT. In addition, we describe a novel case from our dermatology clinic of BP following HSCT in a patient with chronic lymphocytic leukemia (CLL) that transformed to diffuse large B-cell lymphoma (DLBCL).

## 2. Materials and Methods

### 2.1. Systematic Review

The systematic review was conducted in accordance with the Preferred Reporting Items for Systematic Reviews and Meta-Analyses (PRISMA) guidelines and was prospectively registered in the PROSPERO database (Registration No. CRD420251021202). A PRISMA flow diagram summarizing the study selection process is provided in the Supplementary Material (Figure S1).

### 2.2. Search Strategy

A comprehensive database search was performed independently using PubMed, Google Scholar, ScienceDirect, and the ongoing trials registry of the US National Institutes of Health (www.clinicaltrials.gov). The following search criteria were used: (“Bullous Pemphigoid” OR “Pemphigoid”) AND (“Bone Marrow Transplantation” OR “Hematopoietic Stem Cell Transplantation”) AND (“Graft versus Host Disease” OR GvHD), in [MeSH terms] OR [All fields]. Reference lists from the included studies were manually scanned to identify any additional results.

### 2.3. Eligibility Criteria

The eligibility criteria were defined as follows: (i) relevance—case reports of any design describing patients with BP following HSCT and (ii) participants—patients of all ages and sex with a clinical diagnosis of BP following HSCT.

Included were studies of any design that described at least one case of BP following HSCT from which relevant data were extracted. Studies of any language were eligible for inclusion, and non-English reports were translated by the authors when relevant. No restrictions were applied regarding patient age; both adult and pediatric cases were included, provided they met the diagnostic criteria for BP following HSCT.

### 2.4. Data Extraction and Quality

Two reviewers (SGL and AA) independently screened the titles and abstracts of all retrieved articles and subsequently examined the full texts of those considered potentially eligible. Data extraction onto an electronic form was executed by one reviewer (SGL) and validated by the other (AA). The extracted data included the first author’s name, the year of publication, the number of patients, patient age, sex and characteristics, the duration of cutaneous symptoms, the time from HSCT to cutaneous symptoms onset, diagnostic workup, and treatment.

Given that the included studies were primarily case reports or small case series, formal risk of bias tools (e.g., ROBINS-I or GRADE) were not applicable. However, we assessed each report’s diagnostic rigor by confirming the presence of clinical, histological, and immunological findings consistent with BP and by considering the completeness of the reported data.

### 2.5. Statistical Analysis

The data were analyzed using descriptive statistics. Continuous variables were expressed as prevalence and percentages.

## 3. Index Case

In February 2022, a 47-year-old male with no significant family history presented to a tertiary dermatology clinic with a 2-month history of an extensive, painful, pruritic rash involving the scalp, face, torso, and limbs.

His medical history was remarkable for CLL diagnosed in December 2016 and treated with ibrutinib. In June 2019, the disease transformed into DLBCL and was treated with the R-CHOP (rituximab, cyclophosphamide, doxorubicin, vincristine, and prednisone) regimen. Three months later, the patient received an allogeneic bone marrow transplant from his sister (100% HLA match). GvHD developed in June 2020, initially involving the gastrointestinal tract (diarrhea) and liver (elevated bilirubin). Additional symptoms, including recurrent conjunctivitis, sinusitis, dysphagia, and hoarseness, along with oral findings of restricted mouth opening, bilateral buccal reticular white lacing, vesicles, erosions, and sloughing, supported the diagnosis of mucosal GvHD involving the ocular, oral, nasal, and laryngeal sites. No cutaneous involvement was observed at that time.

**Figure 1:**
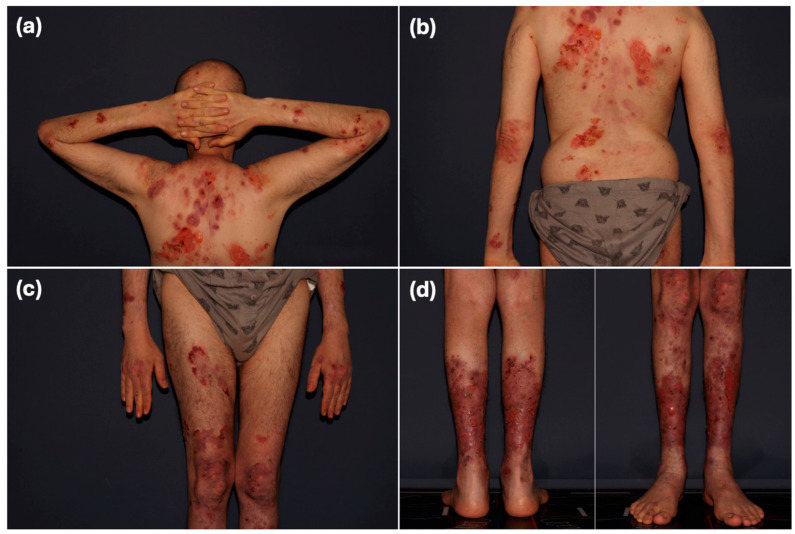
Physical exam of the index case as he first presented to our clinic. (**a**,**b**) Extensive erythematous erosive plaques involving most of the back surface and upper limbs. (**c**,**d**) Extensive erythematous erosive plaques involving the lower limbs. Lesions measured approximately 5–10 cm in diameter. Note: Scale bars are not included in this figure.

In January 2022, 1.5 years after transplantation, when he was stable and afebrile, the patient experienced the sudden onset of a new, widespread rash on the scalp, face, torso, and limbs. It consisted of tense bullae filled with clear or hemorrhagic fluid along with erosions imposed on erythematous plaques or normal-appearing skin (Figure 1).

The patient did not receive any vaccines in the months preceding the onset of the bullous eruption. No temporal or causal association was identified between the patient’s medications and the rash.

No significant laboratory abnormalities were noted. A punch biopsy from the edge of an erosion on the shin showed subepidermal separation with a dermal infiltrate composed of eosinophils, lymphocytes, and neutrophils. The DIF of uninvolved perilesional skin showed linear deposits of IgG and C3 along the dermo-epidermal junction. IIF and ELISA were negative. The BIOCHIP mosaic-based IIF test was negative for the collagen VII antigen but showed dermal deposition of IgG on a salt-split substrate. Based on the correlation of the clinical, pathological, and immunofluorescence findings, a diagnosis of BP was made.

The patient was initially treated with standard therapeutic regimens for BP and/or GvHD. Twice daily total body application of an ultrapotent topical steroid (clobetasol propionate) for BP failed to improve symptoms. Many subsequent treatments were attempted over the years, all of which were discontinued for various reasons: Systemic prednisone [a maximal dose of 40 mg/day (equivalent to 0.8 mg/kg/day)] led to glaucoma and eventually vision loss in the left eye. Minocycline/doxycycline proved ineffective and raised concerns of exacerbating GvHD-related esophagitis. Dapsone led to worsening anemia. Intravenous immunoglobulin (2 g/kg/day) caused significant hemolysis. Methotrexate (5 mg/wk) dramatically increased liver enzyme levels.

GvHD-approved treatments were also associated with serious side effects or proved ineffective. Ruxolitinib, a Janus kinase 1/2 inhibitor (5 mg twice daily), led to multiple hospitalizations for systemic infections, including near-lethal pneumonia, and belumosudil, a rho-associated coiled-coil-containing protein kinase 2 (ROCK-2) pathway inhibitor (200 mg/day), caused severe hypophosphatemia. Extracorporeal photopheresis did not lead to the desired improvement.

By March 2024, nearly 2 years after his initial presentation, the patient’s severe cutaneous condition worsened, resulting in another hospitalization. Given the failure of the many previous treatments, we opted for severe refractory BP therapies. The patient received two doses of rituximab at 375 mg/m^2^ and underwent five rounds of plasma exchange once a week. Additionally, prompted by emerging evidence from the Autoimmune Disease Early Intervention and Prevention Trial (ADEPT) of the effectiveness of dupilumab, a monoclonal antibody targeting interleukin (IL)-4 and IL-13, for moderate–severe BP (20% vs. 4% for placebo, *p* = 0.114) [9], we administered a loading dose of subcutaneous dupilumab at 600 mg.

**Figure 2:**
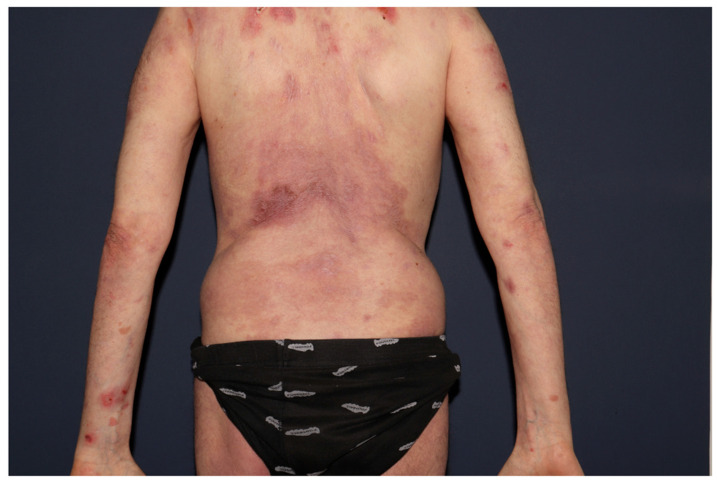
Near-complete re-epithelialization of the extensive cutaneous erosions on the index patient’s back, six weeks after initiating rituximab, dupilumab, and prednisone (August 2024).

Concurrent weekly plasma exchange with dupilumab at a dose of 300 mg every 2 weeks was ineffective. It was only in August 2024, when under this regimen, rituximab at a dose of 375 mg/m^2^ once a week (3 doses) was readministered with prednisone 40 mg/day, that the re-epithelialization of the existing erosions occurred, and no new lesions developed (Figure 2). Disease control has been maintained as of the time of writing this article.

Although the patient underwent standard HLA typing as part of the transplantation protocol, no specific testing was performed to identify HLA alleles previously associated with bullous pemphigoid, such as HLA-DQB1*03:01 [10]. Therefore, it remains unclear whether genetic predisposition contributed to the development of BP in this case.

This is the only reported case of post-HSCT GvHD in a patient with CLL transforming to DLBCL who was diagnosed with severe and recalcitrant new-onset BP. The patient was refractory to multiple standard and advanced treatment modalities administered for the dual morbidity of BP and GvHD. This required a personalized treatment approach that combined several therapies to achieve disease control. This unique case prompted the present in-depth literature review and highlighted the need for a comprehensive systematic review on these combined morbidities.

## 4. Results

The literature search yielded 15 cases, all extracted from case reports or case series dating back to 1986 [6,11,12,13,14,15,16,17,18,19,20,21,22,23,24]. The data were collected and aggregated along with the data from the present case (no. 16). Table 1 summarizes their main characteristics.

There were 12 male and 4 female patients with a mean age of 38 years (range 5–67) at BP diagnosis. Six patients were of Japanese origin. The most common initial hematological disease was acute lymphocytic lymphoma or acute myeloid lymphoma, with four patients each. Fourteen patients presented with GvHD following HSCT; in two cases, GvHD was not mentioned [21,22]. The average time from HSCT to the onset of BP was one year.

### 4.1. Diagnosis

Table 2 summarizes the diagnostic evaluation for BP. A typical clinical presentation of BP was recorded in 13 of the 15 reported patients and in the index patient (no. 16). Further diagnostic workup was available for 13/16 patients. All but one had histological findings characteristic of BP. One patient had histological features of both BP and GvHD (dyskeratotic keratinocytes). All cases but one had a positive DIF test that confirmed BP. The one patient without a DIF test had a positive IIF test but did not undergo salt-split testing. Among the remaining patients, nine had positive IIF–salt-split tests, with antibody localization observed on the epidermal (*n* = 5), dermal (*n* = 2), or both sides (*n* = 2). ELISA or immunoblotting results were positive in 10 patients and negative in 2.

### 4.2. Treatment

Table 3 summarizes the systemic treatments administered for BP. Twelve of the sixteen patients (75%) achieved complete remission (CR), of whom four responded within one month of treatment initiation; seven (58%) achieved CR with prednisolone alone. Two patients (12.5%), including the index case, had refractory disease, and two patients (12.5%) died, one from sepsis and the other from disease progression.

## 5. Discussion

This systematic review raises the awareness of the rare comorbidity of BP and GvHD. Of the 15 reported patients, 13 had chronic GvHD. BP developed in all of them 3 to 30 months after HSCT. These data suggest a potential causal link between GvHD and BP in the post-transplant setting [25,26]. We also added a complex case from our dermatology clinic of a patient with CLL that transformed to DLBCL prior to the development of HSCT-related GvHD, followed 2 years later by BP that was refractory to all standard treatments.

Chronic GvHD is characterized by multi-organ fibrosis and autoantibody production [24,27]. It typically manifests with skin lesions resembling lichen planus or scleroderma [28]. Although GvHD, mostly in the acute setting, can cause subepidermal blisters through basal cell degeneration [4], its association with BP is exceedingly rare [6,11,12,13,14,15,16,17,18,19,20,21,22,23,24]. The pathophysiology underlying the association between HSCT, GvHD, and BP is complex and not fully understood. The current hypothesis suggests that the GvHD-induced basal epidermal damage may expose basement membrane zone (BMZ) proteins, potentially trigger antigen presentation and autoantibody production [6,18]. CD4+ T cells recognize these antigens via major histocompatibility complex-II (MHC-II) on antigen-presenting cells or keratinocytes [29]. In response, they release cytokines that recruit CD8+ cytotoxic T cells [30,31], further exposing BMZ antigens and promoting BP development. This immunological cascade may resemble the phenomenon of epitope spreading, as described in other autoimmune bullous diseases such as BP associated with psoriasis [32]. In this model, initial immune responses to exposed autoantigens may gradually expand to include additional, secondary targets, further amplifying the disease process. Such a mechanism could potentially explain the sequential development of GvHD followed by BP in our and other reported cases. Additionally, GvHD and its treatments (prednisolone and cyclosporine) may disrupt immune homeostasis, altering the Th1/Th2 balance and regulatory T-cell function and fostering autoimmunity [18]. In chronic GvHD, B-cell dysregulation, including heightened B-cell receptor signaling and survival, may further drive the production of autoantibodies against BMZ proteins, such as BP180 and BP230 [26,33,34,35,36]. This immune imbalance is characterized not only by Th1/Th17 skewing but also by enhanced Th2 cytokine expression, which plays a well-established role in BP pathogenesis [37]. Specifically, IL-4 and IL-13 are central to this process: they promote class-switching of B cells toward the IgG4 and IgE isotypes and enhance eosinophilic infiltration [38], both hallmark features of BP [39]. In the GVHD milieu, elevated Th2 cytokines may act synergistically with tissue damage to amplify the autoimmune cascade [40]. Although dupilumab monotherapy did not yield clinical improvement, disease control was ultimately achieved when it was combined with rituximab and plasma exchange. This observation supports a contributory role for Th2 cytokines (IL-4/IL-13) in the pathogenesis of post-HSCT BP, although they likely represent only one component of broader, multifactorial immune dysregulation.

Moreover, chronic GvHD has been linked to profound B-cell dysregulation, including increased autoreactive B-cell survival and antibody production—a key feature in BP development. This dysregulation includes the expansion of autoreactive B-cell clones capable of bypassing central and peripheral tolerance [33]. These clones produce pathogenic autoantibodies against hemidesmosomal components, particularly BP180 and BP230, which are central to BP pathogenesis [1].

The diagnosis of BP in the reported patients with GvHD was well founded due to the distinct histological and immunopathological features of each condition. Chronic GvHD is characterized histologically by hyperkeratosis, hypergranulosis, acanthosis, apoptotic or necrotic keratinocytes, and sub-epidermal splitting. The mainly lymphocytic infiltrate, in any pattern (dermal, epidermal, or both), sometimes extends to the subcutaneous tissue. DIF is positive in up to 86% of patients, exhibiting variable IgM and IgG deposition in a granular and/or linear pattern within the epidermis, BMZ, or upper dermis [41,42,43]. In contrast, BP has a distinct and uniform DIF pattern, with linear IgG ± C3 deposition along the BMZ [1].

Of the 13/16 patients with diagnostic data, all but 1 [6] had a positive DIF test that confirmed BP. This same patient [6] also had a positive IIF test but did not undergo salt-split testing. Among the remaining patients, nine had positive IIF–salt-split tests, with antibody localization observed on the epidermal (n = 5 [13,14,15,19,20]), dermal (n = 2 [24] and the index case), or both sides (n = 2 [18,23]). ELISA or immunoblotting results were positive in 10 patients and negative in 2 ([19] and the index case).

The index case represents a diagnostically unique instance of BP following HSCT. While a bullous form of chronic GvHD (histopathological necrotic keratinocytes) has been described [28,44], the histological findings in this case corresponded with BP. The DIF results were compatible with BP, but IIF and ELISA were negative. In addition, BIOCHIP IIF was negative for collagen VII but positive for dermal deposition of IgG on a salt-split substrate. Although these findings may not fully align with BP criteria [1], IIF negativity occurs in up to 30% of BP cases [45], and the absence of BP-specific antibodies after HSCT has been previously reported [19]. Ultimately, the diagnosis of BP was supported by the characteristic clinical presentation, the absence of dyskeratotic keratinocytes, and positive DIF.

Treatment approaches for BP in this context mirror those for idiopathic BP. Systemic corticosteroids are the cornerstone of treatment, often combined with steroid-sparing immunosuppressants [1]. In our review, 75% of patients achieved full remission, with 58% responding to corticosteroids alone. Our case represents the most treatment-refractory post-HSCT BP reported to date, necessitating more aggressive interventions. For refractory idiopathic BP, several advanced therapies have been explored [46]. Plasmapheresis rapidly removes circulating autoantibodies but can lead to rebound after cessation. Rituximab, a monoclonal anti-CD20 antibody, targets B lymphocytes to reduce autoantibody production and may promote long-term remission. Dupilumab, an anti-IL-4 receptor-α monoclonal antibody and IL-4 and IL-13 blocker, downregulates Th2 responses implicated in BP pathogenesis [46].

Despite its promise, dupilumab alone did not achieve adequate disease control in our case. As such, emerging therapies such as IL-17 and IL-23 inhibitors, may offer alternative avenues for treatment [46]. These agents modulate distinct immunologic pathways implicated in both BP and chronic GVHD and merit further exploration, particularly in refractory cases.

Our multi-pronged approach combining these therapies led to clinical improvement and disease control and may represent a potential treatment strategy for managing refractory BP, both post-HSCT and idiopathic.

Notably, JAK inhibitors such as ruxolitinib have demonstrated efficacy in chronic GvHD, although their role in treating BP remains unclear [47]. In our case, ruxolitinib was attempted but discontinued due to serious infections, emphasizing the delicate balance required in managing overlapping GvHD and BP pathologies.

Importantly, BP is not the only autoimmune bullous disease reported to follow HSCT. Cases of pemphigus foliaceus [48], epidermolysis bullosa acquisita [6,49,50], mucous membrane pemphigoid [51,52,53,54,55,56,57], and linear IgA dermatosis [58] have been documented. Large-scale studies, such as those by Kasperkiewicz et al. [8], indicated an increased but still low overall risk of autoimmune bullous disease post-transplantation. The study by Hofmann et al. [6] further elucidated the immunological landscape, demonstrating a higher frequency of circulating anti-BMZ antibodies, particularly those targeting BP180, in patients with GvHD.

The consistent association of BP with chronic GvHD, as well as its average onset of one year after transplantation, suggests an increased susceptibility in these patients. Interestingly, the mean age of patients in this review (38 years) is substantially lower than the typical age of idiopathic BP, which usually affects elderly individuals. This age discrepancy suggests that BP following HSCT may arise through distinct pathophysiological mechanisms, possibly involving extrinsic post-transplant immune dysregulation rather than intrinsic age-related factors. While BP following HSCT shares clinical and immunopathological features with classic BP, its distinct age distribution and strong association with chronic GvHD may suggest a unique transplant-associated subtype.

Compared to classic BP, which predominantly affects individuals over the age of 70 [1], is associated with HLA-DQB1*03:01 [10], and has a strong IgG4 autoantibody profile [59], post-HSCT BP appears in significantly younger patients (a mean age of 38 in our cohort), with variable or negative serological profiles and unclear HLA associations. These differences suggest distinct immunological drivers, supporting the hypothesis of a “transplant-associated subtype” of BP. However, further studies are needed to validate this classification.

As previously noted, our case represents the most resistant form of BP reported to date, underscoring the importance of a multidisciplinary approach in managing complex presentations.

Looking ahead, several avenues for future research emerge. Identifying predictive biomarkers for BP risk in HSCT recipients would make early intervention possible. Comprehensive immunophenotyping before and after HSCT, during GvHD, and at BP onset could provide crucial insights into the immunopathological cascade. Given the rarity of post-HSCT BP, multi-center collaborations and registries will be vital for accumulating sufficient data to guide evidence-based management.

### Limitations

This review is limited by the small number of reported cases and potential publication bias inherent in case report studies. Furthermore, heterogeneity in diagnostic criteria, treatment reporting, and visual documentation, such as the absence of calibrated scale bars in published images, limits the generalizability and reproducibility of the findings. The retrospective nature and the lack of control groups in included studies further restrict conclusions regarding causality or standardized treatment approaches.

## 6. Conclusions

The study of post-HSCT BP offers valuable insights into the complex interplay among transplantation, GvHD, and autoimmunity. Furthermore, our successful management of a refractory case underscores the importance of an innovative, multidisciplinary approach. As our understanding deepens, we may uncover new therapeutic targets and strategies benefiting not only patients with post-HSCT BP patients but also those with other autoimmune bullous diseases. Given the possible predisposition of patients with GVHD for BP, future research should focus on developing predictive biomarkers and conducting immunophenotyping to better understand the immunopathological mechanisms. The rarity of these cases highlights the need for multi-center collaborations to gather sufficient data for evidence-based management.

## Figures and Tables

**Table 1:** Main characteristics of 16 patients with GvHD/BP after HSCT.

Case	Author, Year	Age at BP Dx (yr)/Sex	Ethnicity *	Initial Hematological Disease	Donor for HSCT	GvHD/BP	Time from HSCT to BP Onset (mo)	Ref.
1	Ueda et al., 1986	46/M	Japanese	AML	Brother	+/+	3	[11]
2	Lacour et al., 1988	29/M	Unknown	AML	Unknown	+/+	10	[12]
3	Delbaldo et al., 1992	47/M	Unknown	CML	Unknown	+/+	3	[13]
4	Kikuchi, 1999	50/M	Unknown	AML	Unknown	+/+	7.5	[14]
5	Szabolcs et al., 2002	18/M	Unknown	ALL	Anonymous	+/+	7	[15]
6	Nagai et al., 2004	40/M	Japanese	MDS	Unknown	+/+	16	[16]
7	Kawasuji et al., 2004	44/F	Unknown	NHL	Unknown	+/+	16	[17]
8	Izumi et al., 2007	55/F	Japanese	ALL	Anonymous	+/+	30	[18]
9	Hofmann et al., 2010	43/M	Unknown	BLL	Unrelated female	+/+	4	[6]
10	Yoneda et al., 2014	57/F	Japanese	AML	Sister	+/+	4	[19]
11	Kato et al., 2015	16/M	Japanese	ALL	Brother	+/+	5	[20]
12	Haber et al., 2017	5/M	Unknown	CMML	Unknown	−/+	8.5	[21]
13	Tamazian and Simpson, 2020	62/M	Filipino	CLL	Unknown	−/+	Unknown	[22]
14	Kawashima et al., 2021	65/M	Japanese	NHL	Unknown	+/+	16.5	[23]
15	Hida et al., 2023	48/F	Unknown	ALL	Unknown	+/+	30	[24]
16	Glazer Levavi et al., 2025	47/M	Ashkenazi Jewish	CLL, DLBCL	Sister	+/+	18	Present study

* Ethnicity was recorded as reported in the original case report; no genetic confirmation was performed. BP, bullous pemphigoid; Dx, diagnosis; HSCT, hematopoietic stem cell transplantation; GvHD, graft-versus-host disease; AML, acute myeloid leukemia; CML, chronic myelogenous leukemia; ALL, acute lymphocytic leukemia; MDS, myelodysplastic syndrome; NHL, non-Hodgkin’s lymphoma; BLL, B-lymphocytic lymphoma CMML, chronic myelomonocytic leukemia; CLL, chronic lymphocytic leukemia; DLBCL, diffuse large B-cell lymphoma.

**Table 2:** Diagnostic evaluation of included cases.

Case	Author, Year	Clinical Findings	Histology	DIF	Circulating Autoantibodies	Donor Autoantibody Testing	Other	Ref.
1	Ueda et al., 1986	Widespread bullous lesions in various stages; mucosal involvement	Sub-basilar bullae; no associated necrotic changes in basal layers, the epidermis, or skin appendages	IgG + C3 deposition along the BMZ	IIF: Circulating IgG antibodies bound with the BMZ of guinea pigs (1:40)	Antibodies not detected (method not mentioned)		[11]
2	Lacour et al., 1988							[12]
3	Delbaldo et al., 1992	Bullous lesions on legs, foot, wrists, elbows, hard palate	Subepidermal splitting; no necrosis; well-preserved epidermis at the roof of bullae; papillary configuration of dermis maintained; bullae devoid of inflammatory cells; a few perivascular lymphocytes in the dermis	Linear IgG + C3 deposition along BMZ; bullous lesions and normal skin	IIF with salt split: Circulating IgG antibodies bound to the epidermal side (>1:50)	IIF and WB: No reactivity	WB: Serum reacted with a 180 kD protein	[13]
4	Kikuchi, 1999	No description (“blister from forearm”)	Subepidermal cleft with eosinophilic infiltration	Linear IgG + C3 deposition along the BMZ	IIF with salt split: Circulating IgG antibodies bound to the epidermal side		WB: Serum reacted with 230 kD and 180 kD proteins	[14]
5	Szabolcs et al., 2002	Pruritic hemorrhagic bullae over the entire body	Subepidermal blister with spongiosis; hemorrhagic infiltration of lymphocytes and eosinophils	Linear IgG + C3 deposition along the BMZ	IIF with salt split: Circulating IgG antibodies bound to the epidermal side		Immunoprecipitation: IgG with strong reactivity to BP180 and faint reactivity to BP230	[15]
6	Nagai et al., 2004	Erythema and tense blisters appeared on the trunk	Consistent with BP; no description	Consistent with BP; no description			Immunoblotting: Consistent with BP; no description	[16]
7	Kawasuji et al., 2004							[17]
8	Izumi et al., 2007	Erythema on extremities, tense blisters on the trunk and extremities, and erosions on the palate and tongue while the conjunctivae were normal	Subepidermal blistering; marked lymphocytic infiltrate with eosinophils in the blister and upper dermis	Linear IgG deposition along the BMZ	IIF with salt split: Circulating IgG antibodies bound to the epidermal and dermal sides (1:320)		ELISA: IgG antibodies against BP180	[18]
9	Hofmann et al. 2010	Blisters on the trunk and extremities	Biopsy specimen not obtained at the time of skin blistering		IIF: High-titer antibodies against BP180		ELISA with immunoblotting: Antibodies against BP180	[6]
10	Yoneda et al., 2014	Erythema and blisters over the entire body	Subepidermal blistering; moderate infiltrates of eosinophils and lymphocytes in the dermis and bullae; dyskeratotic keratinocytes at the roof of bullae with spongiosis	Linear IgG deposition along the BMZ	IIF with salt split: Circulating IgG antibodies bound to the epidermal side	IIF and ELISA: Negative	ELISA: Negative	[19]
11	Kato et al., 2015	Tight blisters on the trunk	Subepidermal cleft with eosinophilic infiltration	Linear IgG deposition along the BMZ	IIF with salt split: Circulating IgG autobodies bound to the epidermal side		Immunoblotting: IgG antibodies against BP180 and BP230	[20]
12	Haber et al., 2017	Blisters						[21]
13	Tamazian and Simpson, 2020	Urticarial plaques on the arms and trunk with peripheral vesicles and central hyperpigmentation; shallow erosions on buccal mucosa	Consistent with BP; no description	Consistent with BP; no description			ELISA: Consistent with BP; no description	[22]
14	Kawashima et al., 2021	Tense blisters on the trunk and both legs	Subepidermal blistering; eosinophil-predominant inflammatory cell infiltration in the upper dermis	IgG deposition along the BMZ	IIF with salt split: Circulating IgG antibodies bound to the epidermal and dermal sides		Immunoblotting: IgG reacted with BP180 and the laminin a3 subunit of laminin 332	[23]
15	Hida et al., 2023	Blisters on hands and legs	Subepidermal blistering with eosinophilic infiltrates	Linear IgG deposition along the BMZ	IIF combined with salt split: Circulating IgG antibodies bound to the dermal side (1:40)	Chimerism analysis: donor-derived B lymphocytes produced laminin 332 AAbs	Immunoblotting: IgG reacted with 140 kda and the b3 subunit of laminin 332	[24]
16	Glazer Levavi et al., 2025	Widespread rash consisting of tense clear/hemorrhagic fluid bullae and extensive erosions, some with sero-hemorrhagic crusts on the face, scalp, trunk, and limbs	Subepidermal separation with dermal infiltrate composed of eosinophils, lymphocytes, and neutrophils	Linear IgG + C3 deposition along the BMZ	Negative IIF BIOCHIP mosaic-based IIF: dermal deposition of IgG on a salt-split substrate		ELISA: Negative	Present study

DIF, direct immunofluorescence; IgG, immunoglobulin G; C3, complement component 3; IIF, indirect immunofluorescence; BMZ, basement membrane zone; BP, bullous pemphigoid; WB, Western blot; ELISA, enzyme-linked immunosorbent assay.

**Table 3:** Systemic treatment received for BP by all included cases.

Case	Author, Year	Systemic Treatment for BP	Outcome	Ref.
1	Ueda et al., 1986	Prednisolone 1 mg/kg/day	CR within one month	[11]
2	Lacour et al., 1988	Prednisolone (dosage unknown)	CR	[12]
3	Delbaldo et al., 1992	Prednisolone and cyclosporine (dosage unknown)	CR	[13]
4	Kikuchi, 1999	Prednisolone (dosage unknown)	CR	[14]
5	Szabolcs et al., 2002	Prednisolone 2 mg/kg/day with mycophenolate, rituximab 375 mg/4 doses, and decalizumab 1 mg/kg/day once a week	CR within one month	[15]
6	Nagai et al., 2004	Prednisolone 50 mg/day, cyclosporine 300 mg/day, minocycline (dosage unknown), and nicotinamide (dosage unknown)	Death (sepsis)	[16]
7	Kawasuji et al., 2004	Prednisolone (dosage unknown)	Refractory disease	[17]
8	Izumi et al., 2007	Prednisolone 40 mg/day	CR	[18]
9	Hofmann et al. 2010	Prednisone 20 mg/day	CR	[6]
10	Yoneda et al., 2014	Prednisolone 1 mg/kg/day	CR within one month	[19]
11	Kato et al., 2015	Prednisolone 1.2 mg/kg/day	Death (disease progression)	[20]
12	Haber et al., 2017	Prednisolone (dosage unknown)	CR	[21]
13	Tamazian and Simpson, 2020	Prednisone, doxycycline, and mycophenolate (dosages are unknown)	CR	[22]
14	Kawashima et al., 2021	Prednisolone 7 mg/day and cyclosporine 50 mg/day	CR	[23]
15	Hida et al., 2023	Tetracycline 1000 mg/day and niacinamide 600 mg/day	CR within one month	[24]
16	Glazer Levavi et al., 2025	Prednisone up to 40 mg/day, dapsone 100 mg/twice a day, doxycycline 100 mg/twice a day, IVIG up to 2 g/kg, MTX 5 mg/week, and mycophenolate 2 g/day A combination of rituximab 375 mg/m^2^ once a week for 5 doses (intermittent administration), plasma exchange once a week, and 300 mg of dupilumab twice a month.	Refractory disease Partial remission with the final combination treatment	Present study

BP, bullous pemphigoid; MTX, methotrexate; IVIG, intravenous immunoglobulin; CR, complete resolution.

## Data Availability

The data presented in this study are available upon request from the corresponding author due to medical confidentiality and patient privacy regulations.

## Supplementary Materials

The following supporting information can be downloaded at https://www.mdpi.com/article/10.3390/jcm14124068/s1. Figure S1: PRISMA 2020 flow diagram for new systematic reviews which included searches of databases and registers only.

## Abbreviations

The following abbreviations are used in this manuscript:

BMZ	Basement membrane zone
BP	Bullous pemphigoid
CLL	Chronic lymphocytic leukemia
IIF	Indirect immunofluorescence
IgG	Immunoglobulin G
CR	Complete resolution
DIF	Direct immunofluorescence
DLBCL	Diffuse large B-cell lymphoma
ELISA	Enzyme-linked immunosorbent assay
GvHD	Graft-versus-host disease
HLA	Human leukocyte antigens
HSCT	Hematopoietic stem cell transplantation
IIF	Indirect immunofluorescence
