# Supplementary material for: Bullous Pemphigoid as a Manifestation of Graft-Versus-Host Disease Following Allogeneic Hematopoietic Stem Cell Transplantation: A Systematic Review and Report of a Novel Case
Source: J Clin Med. 2025 Jun 9;14(12):4068. doi: 10.3390/jcm14124068 (PMC12194141; doi:10.3390/jcm14124068)

Figure S1: PRISMA 2020 flow diagram for new systematic reviews which included searches of databases and registers only

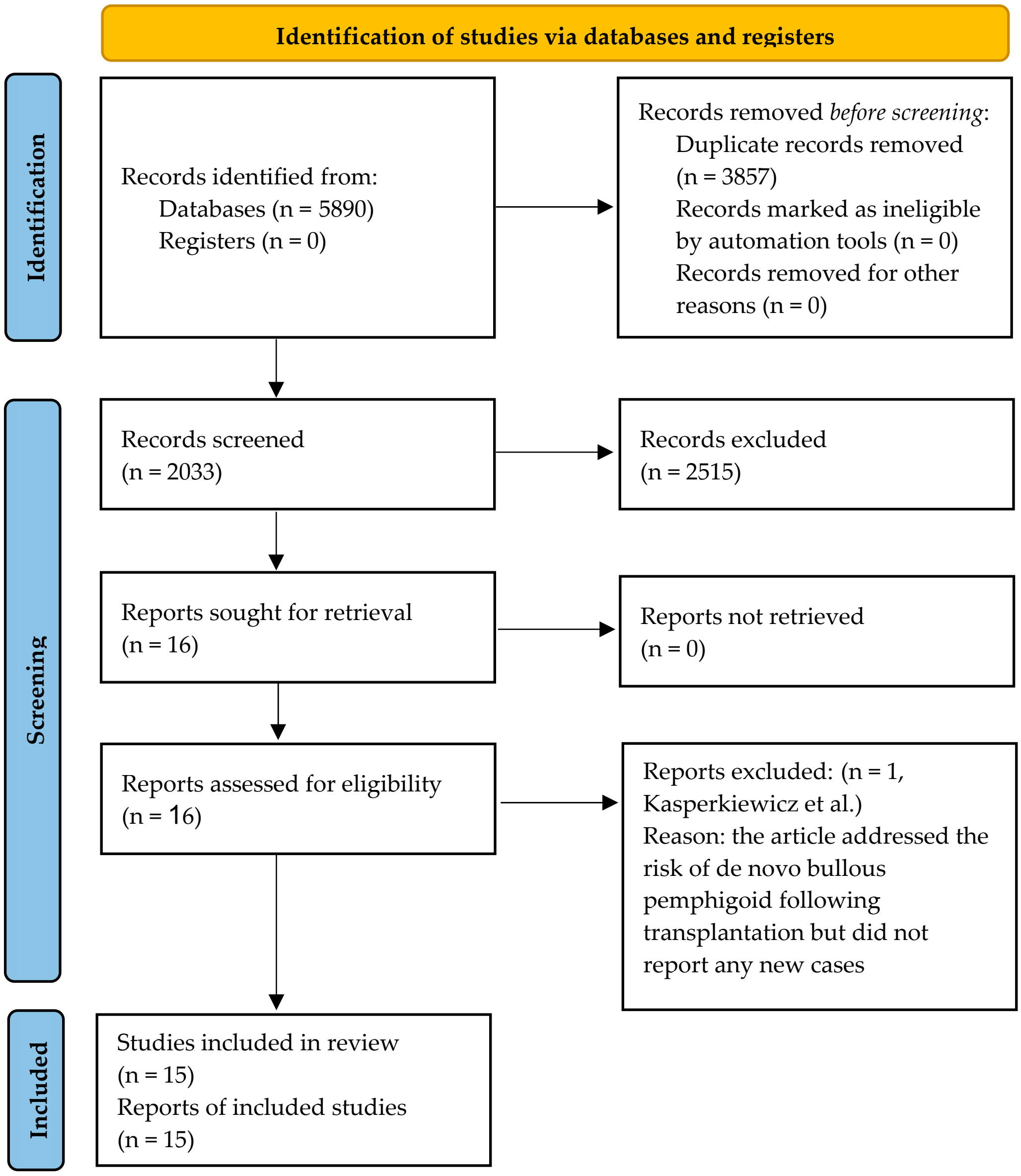

Supplement: Supplementary file 1 [file jcm-14-04068-s001.zip › jcm-3664561-supplementary.pdf]
